# The effects of melatonin and metformin on histological characteristics of the ovary and uterus in letrozole-induced polycystic ovarian syndrome mice: A stereological study

**DOI:** 10.18502/ijrm.v20i11.12365

**Published:** 2022-12-10

**Authors:** Parvin Lohrasbi, Saied Karbalay-Doust, Seyed Mohammad Bagher Tabei, Negar Azarpira, Sanaz Alaee, Bahare Rafiee, Soghra Bahmanpour

**Affiliations:** ^1^Department of Reproductive Biology, School of Advanced Medical Sciences and Technologies, Shiraz University of Medical Sciences, Shiraz, Iran.; ^2^Histomorphometry and Stereology Research Center, Shiraz University of Medical Sciences, Shiraz, Iran.; ^3^Department of Anatomy, School of Medicine, Shiraz University of Medical Sciences, Shiraz, Iran.; ^4^Department of Genetics, Shiraz University of Medical Science, Shiraz, Iran.; ^5^Maternal-Fetal Medicine Research Center, Shiraz University of Medical Sciences, Shiraz, Iran.; ^6^Transplant Research Center, Shiraz University of Medical Science, Shiraz, Iran.

**Keywords:** Polycystic ovarian syndrome, Melatonin, Metformin, Ovary, Uterus, Mice, Stereology.

## Abstract

**Background:**

Polycystic ovarian syndrome (PCOS) with anovulation, hyperandrogenism, ovarian and uterine histological changes, menstrual irregularities, etc. signs is an infertility type. It seems that melatonin and metformin can improve these abnormalities.

**Objective:**

To evaluate the effects of melatonin and metformin on the ovary and uterus in PCOS-induced mice using stereological methods.

**Materials and Methods:**

Seventy-two adult female BALB/c mice (8-wk-old, 20-30 gr) were randomly divided into control (distilled water, gavage), PCOS (90 µg/kg letrozole, gavage), PCOS+metformin (500 mg/kg, gavage), PCOS+melatonin (10 mg/kg, intraperitoneal injection), and PCOS+melatonin control (0.5% ethanol saline) groups (n = 12/each). Another PCOS group was kept for a month to ensure that PCOS features remained. Finally, a stereological evaluation of the uterus and ovary was carried out, and vaginal cytology and serum testosterone levels were assessed.

**Results:**

PCOS mice treated with metformin and melatonin had lower testosterone levels, body weight, and more regular estrus cycles than the PCOS group (p ≤ 0.001). A significant decrease in conglomerate and daughter gland numbers, and primary, secondary, atretic, and cystic follicles numbers with a significant increase in primordial and Graafian follicles, and corpus luteum numbers (p ≤ 0.001) was seen in these treated mice. Also, endometrial vessels' volume and length significantly increased, but ovarian, endometrial, myometrial, stromal, and glands volume, and endometrial and myometrial thickness dramatically declined (p ≤ 0.001).

**Conclusion:**

It appears that metformin and melatonin could restore the PCOS phenotype including estrus cycle irregularity, high testosterone level, and ovarian and uterine micromorphology to the control levels. However, the 2 treatments had similar effects on the examined parameters.

## 1. Introduction

Endocrine problems can leads to the polycystic ovarian syndrome (PCOS), affecting 4-20% of women of reproductive age. PCOS is characterized by at least 2 of 3 criteria: low ovulation or anovulation, hyperandrogenism (HA), and polycystic ovary and/or ovarian volume increase (1). Animal models of PCOS revealed histopathological lesions in the uterus, such as necrosis of stromal mesenchymal cells and lumen epithelial cell hyperplasia (2). In addition, ovaries of PCOS rats showed atretic follicles or degenerating granulosa cells, a double or triple increase of primary, pre-antral, and cystic follicles number, and a decrease in the antral follicles and corpus luteum using the stereology method, which in turn leads to an increase in ovarian size (3, 4).

PCOS has no definitive treatment, but medications such as metformin (N, N-dimethyl biguanide) reduce insulin resistance and androgen levels, improve ovarian morphology and increase mature oocytes in PCOS patients (5). Furthermore, metformin can prevent abnormal endometrial structure development and apoptosis (6). Due to the direct relationship between reducing oxidative stress and increasing oocyte maturation, the use of alternative approaches such as antioxidants has attracted a huge deal of attention today (7). Melatonin (N-acetyl-5-methoxy tryptamine) is a neurohormone synthesized and secreted by the pineal gland, which has antioxidant effects (8). In addition, melatonin has been found in the fluid of the ovarian follicles, which can reduce the damage prompted by oxidative stress in the follicle and thus may increase the fertilization and pregnancy chance in PCOS cases (9), but it needs more exploration. Also, a histological study showed melatonin's ameliorate effect on PCOS mice' ovarian morphology by morphometric method. This led to an increase in the thickness of the granulosa layer and a decrease in the thickness of the theca layer, as well as a decrease in the number of cystic follicles and an increase in the number of corpus luteum (10).

Although 2-dimensional data can be obtained by morphometric methods, stereology-based techniques are preferred. Stereology is a 3-dimensional interpretation of 2-dimensional sections of a substance or tissue that uses random and systematic sampling rather than random selection of specimens; therefore, each part of the area to be studied has the same probability of selection (11).

Given the above issues and using the morphometric method to assay structural changes in most research, this study aims to investigate the effects of metformin (as a common PCOS treatment) and melatonin (as a new treatment) on the ovary and uterus in the PCOS mouse model by stereological techniques, sexual cycle, and serum testosterone levels.

## 2. Materials and Methods

**Figure 1:**
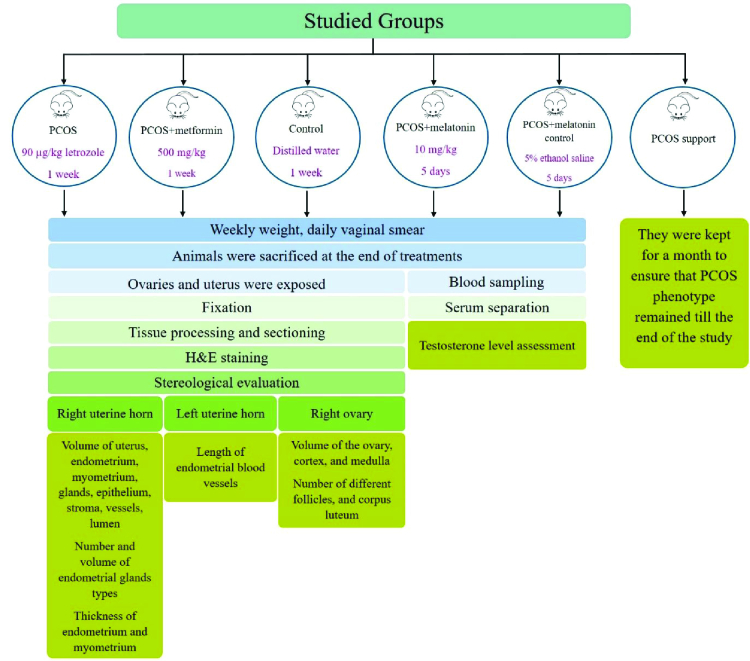
A summary of the work.

A summary of the work is shown in figure 1.

### Animals

The present experimental study was performed on 72 female BALB/c mice without any manipulation (8-wk-old, 20-30 gr). Sample size and randomization were decided based on previous studies (12, 13). Animals were purchased from the Comparative and Experimental Medical Center, Shiraz, Iran. They were kept for 2 wk under standard conditions (22 ± 2 C and 12 hr light/dark cycles) with free access to water and food.

Animals were randomly divided into 6 groups (n = 12/each) and each group was divided into 2 cages (n = 6) as follows:

PCOS: Mice received 0.5 ml of 90 µg/kg letrozole (Abu Raihan pharmaceutical Co., Iran), daily by gavage for 1 wk. Due to the determinate letrozole dose, a pilot study was performed in 4 groups of 8-member each with 60, 90, 120, and 200 µg/kg doses.

PCOS + metformin: PCOS mice received 0.5 mL of 500 mg/kg metformin (Fakher Co., Iran) (6), daily by gavage for 1 wk.

Control: Animals received 0.5 mL of distilled water (as letrozole and metformin solvent) daily by gavage for 2 wk.

PCOS + melatonin: PCOS mice received 100 μl of 10 mg/kg melatonin (Sigma-Aldrich, United States) daily by intraperitoneal injection for 5 days (between 6:00 and 8:00 PM) (10).

PCOS + melatonin control: PCOS mice received 100 μl of 0.5% ethanol saline as a vehicle by intraperitoneal injection for 5 days (between 6:00 and 8:00 PM).

PCOS support: In this group, mice with PCOS were kept for a month without any therapeutic intervention to ensure that letrozole remained effective in inducing PCOS till the end of the study.

### Study design

Weekly weight, daily vaginal smear, serum testosterone levels, and stereological studies of the ovaries and uterus were evaluated to prove PCOS induction in mice and determine the effects of treatments. From the start of the study and grouping of the animals and then conducting experiments and data analysis, one person was unaware of mentioned items to avoid bias.

#### Weekly weight of mice

The animals were weighed once a week from purchase until the end of the study.

#### Determining the sexual cycle

A daily vaginal smear was taken (between 9:00-10:00 AM) for 2 wk to make sure that the animals were mature and had a regular sexual cycle. This process was done to evaluate the effect of drugs on the sexual cycle. Different phases of the sexual cycle (proestrus, estrus, metestrus, and diestrus) were distinguished based on vaginal cells features. For this purpose, vaginal cells were collected, fixed, stained with Giemsa, and observed with an optical microscope.

#### Hormonal assay

The mice were sacrificed, blood was collected and serum isolation was done, and the samples were frozen at -80 C. Then, the serum testosterone level was measured by enzyme-linked immunosorbent assay using a special kit (Monobid, California, USA, measuring range 10-800 ng/dl) according to the manufacturer's instructions.

#### Stereological evaluation of the right ovary and horns of the uterus

#### 2.2.4.1. Preparation of the ovarian tissue 

**Figure 2:**
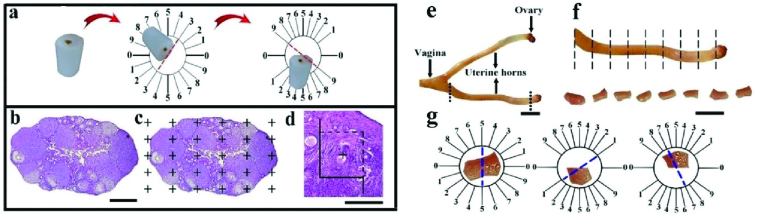
(a) The cylindrical paraffin block of the ovary was sectioned by isotropic uniform random. (b) The ovarian histological sections were stained with Hematoxylin and Eosin (H&E) (Scale bar = 1 mm). (c) The volume of the cortex and medulla were assessed by the point-counting method (Scale bar = 1 mm). (d) The numbers of the different follicles and corpus luteum were assessed by the optical disector technique (scale bar = 100 µm). (e) Anatomy of the mouse uterus (scale bar = 0.7 mm). (f) According to its length, the mouse uterine horn was removed and sectioned into 8-12 parts (scale bar = 0.6 mm). (g) Isotropic uniform random sections were obtained from the mouse uterine horn using the orientation technique.

After blood sampling, ovaries and uterus were removed, and fixed in neutral buffered formalin. The ovary was weighed, processed, and embedded in cylindrical paraffin blocks and sectioned based on isotropic uniform random sections to evaluate the follicles volume by nucleator (Figure 2a). For this purpose, the block of the ovary was randomly put on the φ-clock, and a random number was selected. Then, the block was located on the θ-clock along its cutting surface on the 0-0 axis. This was done randomly for the other block pieces (13). Successive 4 and 25 μm thick sections were obtained and stained with hematoxylin and eosin (H&E) (Figure 2b).

#### 2.2.4.2. Estimationof the volume of the ovary

The total volumes of the ovary were estimated based on the “Cavalieri method” at the final × 52 magnification. For this purpose, 8-12 sections per animal were sampled by systematic random sampling, and the first section was randomly selected; the following sections were achieved at equal intervals. The volume of the ovary was estimated by the “point-counting method” (Figure 2c) and by the following formula: 


$$V\left(ovary\right)=A\times T$$

A=∑P(ovary)×a/p

Where, ∑ P is the total number of points hitting on the figures of the ovary, a/p denotes the area associated to each point, and T represents the interval between the chosen sections (13).

#### 2.2.4.3. Estimation of the volumes of the cortex and medulla

Estimation of the cortex and medulla total volume (4 µm sections) was performed using “point-counting method” (Figure 1) at the final × 52 magnification by the following equations: 


$$Vv\left(structure\right)=\sum P\left(structure\right)/\sum P\left(total\right)$$

V(structure)=Vv(structure)×V(ovary)

∑ P (structure) indicates the total points hitting the selected structure.

#### 2.2.4.4. Estimation of the number of the different follicles and corpus luteum

The “optical disector method” was utilized to calculate the number of primordial, primary, secondary, Graafian, atretic, and cystic follicles, and corpus luteum using the 25 µm sections. The optical disector consists of an “Eclipse microscope (E200, Nikon, Tokyo, Japan)” with a high numerical aperture (NA = 1.30) × 20 objective, connected to a video camera, and an “electronic microcator (MT12, Heidenhain, Germany)”. The unbiased counting frame was used to count the number of the follicles and corpus luteum with a “stereology software (Stereolite, SUMS, Iran)”. The follicles were identified in the good focus of the frame located completely or partially in the counting frame, and did not contact the solid line (Figure 2d). “The numerical density and total number of the follicles and corpus luteum was calculated by the consequent formula: 


$$Nv\left(cells\right)=\sum Q-/(\sum P\times (a/f)\times h)\times (t/BA)$$

$$N\left(total\right)=Nv\times V\left(ovary\right)$$

ΣQ- shows the number of follicles appearing into disector height, ΣP denotes the entire counting frames in all microscopic fields, and h is the disector height. Additionally, a/fand t represent the frame area and the accurate section thickness measured with the microcator, respectively. BAis the block advance of the microtome”. The thickness of the ovary section was measured in the whole microscopic fields with uniform random sampling from every ovary section (14).

#### 2.2.4.5. Preparation of the uterus tissue sections

The right and left uterine horns were separated, and after measuring their weight the primary volume was measured by the immersion method. Then, based on the length of the uterine horn, 8-12 parallel sections were obtained (Figure 2f). The pieces of the right uterine horn were removed for histological processing. For estimating the total length of the vessels, the pieces of the left uterine horn were sectioned by the isotropic uniform random sections method as described elsewhere (13) (Figure 2g). Sections with 4 and 25 μm were obtained from uterine horns tissues and stained with hematoxylin and eosin (H&E) for stereological evaluations.

#### 2.2.4.6. Estimation of the volumes of the uterine structures

The volumes of the lumen, perimetrium, myometrium, and endometrium (epithelium, gland, stroma, and blood vessels) layers from micrometer sections of the right uterine horn were assessed by the “point-counting method” as described before (13). The volume density and total volume of uterine structures was measured by the following formula, respectively: 


$$Vv\left(structure\right)=\sum P\left(structure\right)/\sum P\left(total\right)$$

$$V\left(structure\right)=Vv\left(structure\right)\times V\left(uterinehorn\right)$$

#### 2.2.4.7. Estimation of the length of the blood vessels

The length density of the uterine blood vessels from 4 µm sections was evaluated using an unbiased counting frame previously described and by the following formula: 


Lv(bloodvessel/uterus)=2∑Q/(∑P×a/f)

*∑* Q is the total number of blood vessels whose diameter was more than 10 µm, *∑* P also stands for the number of frame points hitting the uterine tissue, while a/f is the counting frame area at the last × 20 magnification. The total length of blood vessels was estimated by:


$$L\left(bloosvessels\right)=LV\left(bloodvessels/uterus\right)\times V\left(uterinehorn\right).$$

#### 2.2.4.8. Estimation of the number of uterine glands

The number of uterine glands was estimated using the optical disector method described before (14). The following formula assessed the numerical density and total number of the gland, respectively:


$$Nv\left(\mathrm{𝑢𝑡𝑒𝑟𝑖𝑛𝑒}\mathrm{𝑔𝑙𝑎𝑛𝑑}/\mathrm{𝑡𝑜𝑡𝑎𝑙}\right)=\sum Q-/\left(\sum P\times \left(a/f\right)\times h\right)\times \left(t/BA\right)N\left(total\right)=Nv\times V\left(uterinehorn\right)$$

#### 2.2.4.9. Morphology of the endometrial gland

The morphological types of endometrial glands were recognized and categorized to assess hyperplastic or neoplastic changes in the endometrium. The normal endometrial gland is a simple tubular gland with a thin lumen that may be oval, circular, or elongated. The cystic endometrial glands are big and round. A gland endometrial with the daughters refers to glands with irregular shapes and sizes that make up the daughter glands within the epithelium or at the level of the lumen of the mother's glands or external. The conglomerate endometrial gland has adjacent glands without stromal interference, with several lumens joined (16). A video-microscopy system was employed for morphological evaluation of endometrial glands, considering slides of 4 µm thickness sections at a last × 20 magnification in 8-12 random microscopic fields.


Glandtypepercentage=N(glandtype)×100/totalnumber

#### 2.2.4.10. Estimation of the endometrial and myometrial thickness

To assess the endometrial and myometrial thickness, the harmonic mean thickness of the 4 µm sections of mentioned layers was evaluated based on the “orthogonal intercepts method” (17). The probe of stereology (isotropic lines) at the final × 10 magnification was covered the live photos. The distance between the inner and outer surfaces of the aforesaid layers was measured by drawing a vertical line from the outside surface to the touch point of the isotropic line with the inside surface of the structures. The next formula assessed the mean thickness:

*Harmonic mean layer thickness = 8/(3π)× Harmonic mean of orthogonal intercepts*

*The harmonic mean of orthogonal intercepts = number of measurements/Total of the reciprocal of orthogonal intercepts (oi)*

*number of measurements* / $\left[\frac{1}{oi}+\frac{1}{oi}+\frac{1}{oi}+\frac{1}{oi}\cdots \right]$

### Ethical considerations

All animal procedures were approved by the Animal Ethical Committee of Shiraz University of Medical Sciences, Shiraz, Iran (Code: IR.SUMS.REC.1398.619). To reduce discomfort and pain in animals, all steps were performed according to the protocols of the ethics committee.

### Statistical analysis

The obtained data are presented as mean ± S.D. The results were statistically analyzed using GraphPad Prism 9 (GraphPad Software Inc., San Diego, CA) by ANOVA, followed by the Tukey post hoc test to evaluate the difference between the studied parameters in the 3 groups (control, PCOS, and PCOS + metformin). In addition, an independent *t* test was used to determine the differences between PCOS + melatonin and its corresponding control groups, and also PCOS + melatonin and PCOS groups. The first type of error (p-value) was 5%, and the confidence interval (CI) was 95%. Significant differences were considered at p < 0.05.

## 3. Results

Metformin and melatonin as treatment groups reversed and corrected PCOS phenotypes (sexual cycle irregularities, elevated body weight, and testosterone levels, increase in the ovarian and uterine endometrium, myometrium, stroma, epithelium, and glands volume, endometrial and myometrial thickness, atretic, cystic, primary and secondary follicles' number, daughter and conglomerate glands' number, and decrease in primordial, and Graafian follicles, corpus luteum, and normal glands' number, and endometrial vessels' volume and length), and brought them to their control groups' levels.

### Body weight 

Metformin and melatonin reduced the elevated body weight of PCOS mice after the 3 ${}^{\text{rd}}$ wk and brought it to the control groups' levels.

### Determining the estrus cycle 

Metformin and melatonin regulated the sexual cycle and significantly increased proestrus, and estrus phases percentage (p = 0.02, and p = 0.01, respectively, CI = 95%), and decreased diestrus phase percentage (p ≤ 0.001, and p = 0.004, respectively, CI = 95%) compared to the PCOS group.

### Serum testosterone levels

Metformin and melatonin significantly reduced elevated serum testosterone levels in the PCOS mice (p = 0.001, CI = 95%).

### Ovarian stereological results 

#### Volumes of the ovary, cortex, and medulla 

**Figure 3:**
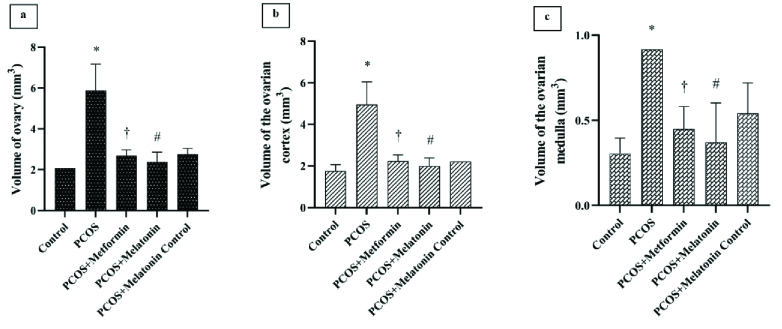
Ovarian (a), Cortex (b), and Medulla (c) volume (mm^3^). *PCOS vs control, † PCOS vs. PCOS + metformin, #PCOS vs. PCOS + melatonin. Data are the Mean ± SD. Statistical analyses were performed by ANOVA followed by Tukey's test for multiple comparisons. All differences were considered significant at p < 0.05 and CI = 95%. PCOS: Polycystic ovarian syndrome.

Metformin and melatonin significantly reduced the increased volume of the ovary, cortex, and medulla compared to the PCOS group (p ≤ 0.001, p ≤ 0.001, and p = 0.03, respectively, CI = 95%) (Figure 3).

#### Numbers of the ovarian follicles and corpus luteum

**Figure 4:**
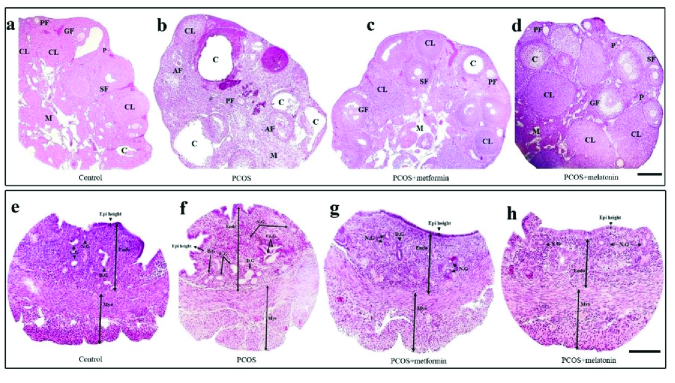
Ovarian (a-d) and Uterine (e-h) histoarchitecture of the control, PCOS, PCOS + metformin, and PCOS + melatonin groups. “a” shows corpus luteum (CL) and follicles at different stages (primordial follicles (P), primary follicle (PF), secondary follicle (SF), and Graafian follicle (GF)). “b” shows the cystic ovary from PCOS mice (cyst (C), atretic follicles (AF)). “c” and “d”, show the ovaries from PCOS mice after treated with metformin and melatonin which have corpus luteum, Graafian follicle, and other pre-antral follicles. M, medulla. “f” shows an increase in endometrial and myometrial thickness (µm), epithelial height (µm), and gland density. “g” and “h” show a reduction in mentioned parameters in “e”. PCOS, polycystic ovarian syndrome, Endo, endometrium, Myo, myometrium, Epi, epithelium, N.G, normal gland, C.G, cystic gland, D.G, daughter gland, Co.G, conglomerate gland. (a-d) Scale bar = 100 µm, and (e-h) scale bar = 200 µm.

Metformin and melatonin significantly increased the declined number of corpus luteum and primordial and Graafian follicles in the PCOS mice, and conversely decreased the elevated number of primary, secondary, atretic, and cystic follicles (Table I, Figure 4).

### Uterine stereological results 

#### Volume of the uterus 

**Figure 5:**
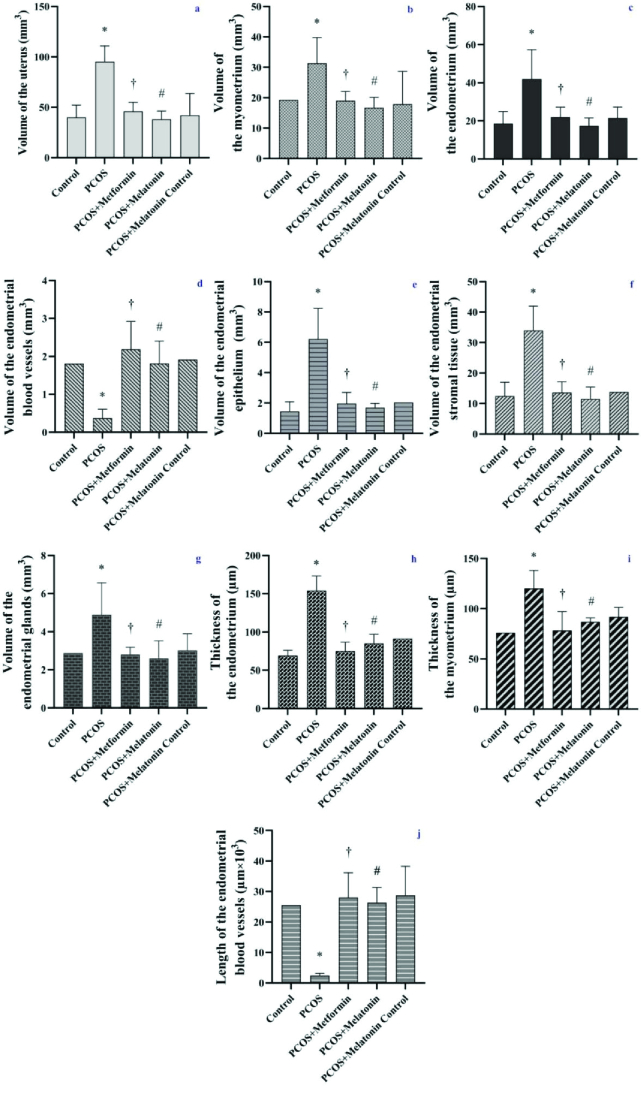
The weight (gr) of uterus (a) and volume (mm^3^) of the uterus (b), myometrium (c), endometrium (d), endometrial vessels (e), the epithelium (f), stroma (g), and gland (h), the thickness of the endometrium (i) and myometrium (j) (µm) and the length of the endometrial vessel (µm × 10^3^) (k) of PCOS and treated mice. *PCOS vs control, † PCOS vs. PCOS + metformin, #PCOS vs. PCOS + melatonin. Data are the Mean ± SD. Statistical analyses were performed by ANOVA followed by Tukey's test for multiple comparisons. All differences were considered significant at p < 0.05 and CI = 95%. PCOS: Polycystic ovarian syndrome.

Metformin and melatonin significantly reduced the uterine volume of the PCOS mice (p ≤ 0.001, CI = 95%) (Figure 5a).

#### Volumes of the endometrium, myometrium, and perimetrium

The volume of myometrium and endometrium in the PCOS mice reduced after treatment with metformin (p = 0.01 and p = 0.02, respectively, CI = 95%), and melatonin (p = 0.01, CI = 95%) (Figure 5b, c). Perimetrium volume did not exhibit a significant difference in any of the study groups.

#### Volumes of the endometrial structures

Metformin and melatonin decreased the volume of the endometrial glands, stroma, and epithelium of PCOS mice (p = 0.03, and p ≤ 0.001, respectively, CI = 95%), and increased their endometrial vessel's volume (p ≤ 0.001, CI = 95%). The lumen volume showed no significant difference inthe studied groups (Figure 5d, e, f, and g).

#### Numbers and volumes of the endometrial gland types

Metformin and melatonin decreased the numbers and volumes of the endometrial total, daughter, and conglomerate glands and increased the normal glands' number and volume in PCOS mice. Cystic glands did not show significant differences in different groups (Table II, Figure 4).

#### Thickness of the endometrium and myometrium

Increased endometrial and myometrial thickness in PCOS mice reduced after treatment with metformin and melatonin (p ≤ 0.001, CI = 95%) (Figure 4 and Figure 5h, i).

#### Length of the endometrial vessels 

Metformin and melatonin increased the endometrial vessels' length of PCOS mice and brought it to their control groups' levels (p ≤ 0.001, CI = 95%) (Figure 5j).

There was no significant difference between the PCOS+melatonin and its corresponding control groups in any study parameters. Also, metformin and melatonin were not significantly different in their effectiveness. Besides, after 1 month, body weight, serum testosterone levels, and continuous diestrus stage in the PCOS support group were the same as the second wk after the beginning of letrozole gavage (p = 0.3, CI = 95%) and so were considered as PCOS range.

**Table 1:** Comparison of the ovarian follicles and corpus luteum number between groups


**Group variable** **(number)**	**Control**	PCOS ${}^{*}$	**PCOS + metformin** ${}^{†}$	**P-value (between control, PCOS, and PCOS + metformin groups)**	**PCOS + melatonin** ${}^{\#}$	**PCOS + melatonin control**	**P-value (between PCOS + melatonin and its control groups)**
**Primordial follicle **	180.3 ± 30.93	78.86 ± 19.43 ${}^{*}$	190.5 ± 47.95 ${}^{†}$	≤ 0.001	188.3 ± 22.73 ${}^{\#}$	164.8 ± 20.09	0.12
**Primary follicle **	93.67 ± 45.18	448.4 ± 84.86 ${}^{*}$	103.2 ± 8.524 ${}^{†}$	≤ 0.001	129.7 ± 23.35 ${}^{\#}$	116.4 ± 34.59	0.50
**Secondary follicle **	11.67 ± 5.14	165.2 ± 35.32 ${}^{*}$	20.41 ± 14.30 ${}^{†}$	≤ 0.001	18.25 ± 5.89 ${}^{\#}$	23.73 ± 7.09	0.22
**Graafian follicle **	52.08 ± 15.26	17.60 ± 4.32 ${}^{*}$	48.11 ± 7.33 ${}^{†}$	≤ 0.001	52.48 ± 15.85 ${}^{\#}$	41.41 ± 17.95	0.33
**Atretic follicle **	25.03 ± 8.26	82.11 ± 14.69 ${}^{*}$	14.75 ± 3.66 ${}^{†}$	≤ 0.001	21.74 ± 6.59 ${}^{\#}$	19.35 ± 4.20	0.51
**Cystic follicle **	23.41 ± 5.98	344.6 ± 67.06 ${}^{*}$	31.19 ± 8.26 ${}^{†}$	≤ 0.001	36.27 ± 7.68 ${}^{\#}$	35.70 ± 8.58	0.91
**Corpus luteum **	113.8 ± 35.41	42.08 ± 11.24 ${}^{*}$	126.5 ± 30.34 ${}^{†}$	≤ 0.001	148.0 ± 32.34 ${}^{\#}$	132.5 ± 23.37	0.41
Data are the Mean ± SD. Statistical analyses were performed by ANOVA followed by Tukey's test for multiple comparisons. All differences were considered significant at p < 0.05 and CI = 95%. *PCOS vs. control, † PCOS vs. PCOS + metformin, #PCOS vs. PCOS + melatonin. PCOS: Polycystic ovarian syndrome							

**Table 2:** Comparison of the glands number and volume between groups


**Group variable**	**Control**	**PCOS** ${}^{*}$	**PCOS + metformin** ${}^{†}$	**P-value (between control, PCOS, and PCOS + metformin groups)**	**PCOS + melatonin** ${}^{\#}$	**PCOS + melatonin control**	**P-value (between PCOS + melatonin and its control groups)**
**Total glands number**	16.40 ± 3.21	36.20 ± 5.50 ${}^{*}$	19.00 ± 3.39 ${}^{†}$	≤ 0.001	17.00 ± 2.82 ${}^{\#}$	16.40 ± 2.41	0.72
**Normal glands** **number**	11.40 ± 3.43	3.80 ± 1.64 ${}^{*}$	12.60 ± 1.67 ${}^{†}$	≤ 0.001	11.80 ± 1.79 ${}^{\#}$	10.60 ± 2.07	0.36
**Cystic glands** **number**	2.60 ± 0.55	3.60 ± 1.14	2.80 ± 1.64	0.40	2.80 ± 1.10	2.60 ± 1.14	0.78
**Daughter glands** **number**	1.40 ± 0.57	14.40 ± 3.98 ${}^{*}$	1.60 ± 0.89 ${}^{†}$	≤ 0.001	1.20 ± 0.45 ${}^{\#}$	1.20 ± 0.45	0.99
**Conglomerate** **glands number**	1.00 ± 00	16.20 ± 2.28 ${}^{*}$	1.2 ± 0.45 ${}^{†}$	≤ 0.001	1.20 ± 0.45 ${}^{\#}$	1.40 ± 0.55	0.55
**Normal glands** **volume (mm^3^)**	2.04 ± 0.62	0.43 ± 0.14 ${}^{*}$	2.17 ± 0.49 ${}^{†}$	≤ 0.001	1.92 ± 0.48 ${}^{\#}$	2.08 ± 0.82	0.71
**Cystic glands** **volume (mm^3^)**	0.47 ± 0.09	0.27 ± 0.16	0.43 ± 0.15	0.09	0.46 ± 0.12	0.55 ± 0.15	0.33
**Daughter glands** **volume (mm^3^)**	0.21 ± 0.18	2.19 ± 0.41 ${}^{*}$	0.23 ± 0.19 ${}^{†}$	≤ 0.001	0.17 ± 0.07 ${}^{\#}$	0.23 ± 0.06	0.18
**Conglomerate** **glands volume (mm^3^)**	0.16 ± 0.06	2.12 ± 0.69 ${}^{*}$	0.21 ± 0.10 ${}^{†}$	≤ 0.001	0.18 ± 0.13 ${}^{\#}$	0.19 ± 0.08	0.91
Data are the Mean ± SD. Statistical analyses were performed by ANOVA followed by Tukey's test for multiple comparisons. All differences were considered significant at p < 0.05 and CI = 95%. *PCOS vs. control, † PCOS vs. PCOS + metformin, #PCOS vs. PCOS + melatonin. PCOS: Polycystic ovarian syndrome							

## 4. Discussion

In this study, induction of PCOS with our new dose of letrozole in mice led to diestrus stage continuity, weight gain, and elevated serum testosterone levels. The volume of the ovaries, cortex, and medulla, the number of primary, secondary, atretic, and cystic follicles increased. Conversely, the number of primordial, and Graafian follicles, and corpus luteum decreased. Uterine, endometrial epithelium, stroma, and glands' volume, endometrial and myometrial thickness, and daughter and conglomerate glands' numbers increased. In contrast, endometrial vascular volume and length, and the number of normal glands decreased. Treatment with metformin and melatonin significantly restored these parameters to their controls' levels.

Mouse models can efficiently simulate the effects of PCOS on humans, and also they are useful in terms of a short reproductive lifespan and reasonable price (18). In previous studies, the duration of letrozole administration in mice was usually longer (50 µg/day, 60, and 35 days) (19, 20). Our new dose of letrozole (90 µg/kg) could induce PCOS in mice in only 7 days. Letrozole, an aromatase inhibitor, prevents the conversion of androgens to estrogen, thus, increasing androgen production. This event disrupts lipid metabolism, and increases insulin levels, and luteinizing hormone (LH) secretion, which stimulate more androgens production, leading to PCOS phenotype (weight gain, cystic ovarian morphology, and sexual cycle irregularity). Also, androgens stimulate theca cells proliferation (21), explaining the elevated PCOS ovarian volume in this study.

Follicular growth disorders in PCOS are likely to be caused by excessive LH/follicle-stimulating hormone ratio, and androgen levels (22). HA is the main reason for follicular arrest, non-selection of a dominant follicle for ovulation, and cyst formation (23).

Androgen receptor is induced in the PCOS uterus, through which androgens can promote endometrial growth (24). Hyperinsulinemia caused by PCOS can inhibit the natural differentiation process of endometrial stroma and reduce the expression of insulin-like growth factor (IGF)-binding proteins, thereby increasing IGF-I levels, which increases cell proliferation and hyperplasia (25). HA can also induce the emergence of various abnormal glands such as conglomerate, which is considered an unfavorable prognosis for precancerous conditions (26). Therefore, hyperinsulinemia, HA, and elevated IGF-I levels may contribute to endometrial dysfunction and hyperplasia. In agreement with our research, a stereological study reported an increase in ovarian and corticomedullary volume, and atretic and small secondary follicles number, and a decrease in primordial, Graafian, antral follicles, and corpus luteum number in letrozole-induced PCOS rats (12). Increased uterine weight, endometrial, myometrial, and stromal thickness under the influence of androgens indicate their trophic effects on the uterus in PCOS rats, which is parallel with this study's results (27).

No studies have addressed changes in vascular volume and length in PCOS and the effect of metformin and melatonin. Women with PCOS have been found to have uterine abnormal blood flow. HA may negatively affect uterine blood flow, and disruption of angiogenic factors such as vascular endothelial growth factor (VEGF) which its expression levels are reduced in PCOS condition and may play an essential role in the pathology of this syndrome (28). These findings may explain the reduction of endometrial vascular length and volume in the PCOS group in our study.

Metformin can reduce hepatic glucose production, inhibit gluconeogenesis and adipogenesis, and improve insulin sensitivity by decreasing androgen, insulin levels, LH secretion, and in some cases, increasing sex hormone-binding globulin, thus decreasing free testosterone (29). In this way, metformin can result in weight loss and reduce the proliferation of androgen-induced ovarian theca cells in PCOS, reducing ovarian volume and partially restoring follicular dynamics (30). Therefore, metformin can be effective in preventing follicular atresia and cyst formation, as well as in improving ovarian folliculogenesis, ovulation induction, and corpus luteum formation in PCOS, which may explain this study results. In accordance with our results, one study determined secondary, cystic and atretic follicles' numbers decrement, and Graafian follicles and corpus luteum increment in PCOS rats (31). Metformin has been reported to improve uterine morphology and endometrial blood flow by reducing insulin, suppressing testosterone in the bloodstream, reducing the level of uterine androgen receptors, and inhibiting inflammation. Also, a decrease in endometrial and epithelial thickness and conglomerate gland's number was reported (6). In line with this study, another research determined that metformin reduces cell viability, induces cellular apoptosis, and leads to uterine volume and weight loss (32).

The effect of melatonin on metabolic and reproductive disorders in PCOS rats, determined that melatonin reduces body weight, body mass index, and insulin levels (25). Another research reported that melatonin could reduce testosterone levels by increasing the testosterone conversion into estrone (33), which is in line with our results. Melatonin also has an antioxidant effect on the corpus luteum, and promotes follicular maturation and ovulation by protecting follicles against oxidative stress and their escape from atresia (34).

In PCOS, the number of atretic follicles increased due to follicular melatonin concentration reduction and oxidative stress amplification, and the resulting follicular damage (9). Consistent with our results, a study found that melatonin increased corpus luteum and antral follicles and decreased ovarian cysts in PCOS rats (35). According to the above explanations, it seems that the antioxidant properties of melatonin can positively affect folliculogenesis.

The effects of melatonin on endometrial tissue and its therapeutic effects in PCOS have not yet been well documented. Melatonin increases aromatase expression and thus decreases androgen production, which causes the reduction of uterus volume. Melatonin can also decrease expression of IGF-I and its receptor in PCOS, suggesting its involvement in improving endometrial hyperplasia (33). In letrozole-induced PCOS hamsters, melatonin reversed endometrial structures such as endometrial glands, hyperplasia, stromal proliferation, androgen receptor expression, and insulin levels, all of which help restore uterine health in PCOS (36). In pinealectomized rats, the increase in endometrial thickness decreased after receiving melatonin. Also, melatonin caused a decrease in conglomerate glands and an increase in normal glands in PCOS rats (37). These findings agree with our results. Besides, melatonin causes different impacts on the regulation of angiogenesis depending on the conditions. Many studies have assigned these effects to the role of melatonin in the regulation of VEGF and its receptors, but its exact mechanisms are still unknown (38). According to the evidence of decreased uterine blood flow and VEGF expression in PCOS (28), it is assumed that increased VEGF expression under the influence of melatonin was effective in increasing the volume and length of endometrial vessels, which of course, requires more investigations.

### Limitations

At the time of the study, there was a time limitation for performing some other tests, including metabolic tests.

## 5. Conclusion

This study demonstrated the potential of melatonin as a therapeutic agent in PCOS like metformin. Considering the beneficial effects of melatonin on the studied parameters in present research (reduction of testosterone, regulation of estrus cycle, improvement of uterine and ovarian morphology and histology), it can be suggested as an alternative treatment to restore the normal function of PCOS-affected tissues. Further investigations are required to confirm these findings through clinical studies.

##  Conflicts of interest

The authors have no conflict of interest to declare.
